# FBP1 inhibits NSCLC stemness by promoting ubiquitination of Notch1 intracellular domain and accelerating degradation

**DOI:** 10.1007/s00018-024-05138-x

**Published:** 2024-02-13

**Authors:** Tianyu He, Yanye Wang, Wang Lv, Yiqing Wang, Xinye Li, Qingyi Zhang, Han-Ming Shen, Jian Hu

**Affiliations:** 1 Department of Thoracic Surgery, The First Affiliated Hospital, Zhejiang University School of Medicine, Hangzhou, China; 2 Department of Thoracic Surgery, Sir Run Run Shaw Hospital, Zhejiang University School of Medicine, Hangzhou, China; 3 Department of Physiology, Yong Loo Lin School of Medicine, National University of Singapore, Singapore, Singapore; 4 Faculty of Health Sciences, University of Macau, Macau, China; 5 Key Laboratory of Clinical Evaluation Technology for Medical Device of Zhejiang Province, Hangzhou, China

**Keywords:** NSCLC, Stemness, FBP1, NICD1, Ubiquitination

## Abstract

The existence of cancer stem cells is widely acknowledged as the underlying cause for the challenging curability and high relapse rates observed in various tumor types, including non-small cell lung cancer (NSCLC). Despite extensive research on numerous therapeutic targets for NSCLC treatment, the strategies to effectively combat NSCLC stemness and achieve a definitive cure are still not well defined. The primary objective of this study was to examine the underlying mechanism through which Fructose-1,6-bisphosphatase 1 (FBP1), a gluconeogenic enzyme, functions as a tumor suppressor to regulate the stemness of NSCLC. Herein, we showed that overexpression of FBP1 led to a decrease in the proportion of CD133-positive cells, weakened tumorigenicity, and decreased expression of stemness factors. FBP1 inhibited the activation of Notch signaling, while it had no impact on the transcription level of Notch 1 intracellular domain (NICD1). Instead, FBP1 interacted with NICD1 and the E3 ubiquitin ligase FBXW7 to facilitate the degradation of NICD1 through the ubiquitin–proteasome pathway, which is independent of the metabolic enzymatic activity of FBP1. The aforementioned studies suggest that targeting the FBP1-FBXW7-NICD1 axis holds promise as a therapeutic approach for addressing the challenges of NSCLC recurrence and drug resistance.

**Supplementary Information:**

The online version contains supplementary material available at 10.1007/s00018-024-05138-x.

## Introduction

Lung cancer is a leading cause of death worldwide [1], the precise mechanisms underlying its development and progression remain elusive. Metabolic reprogramming is a fundamental hallmark of cancer [2, 3], wherein the excessive activation of anaerobic glycolysis and the suppression of gluconeogenesis are notable manifestations [4, 5]. Fructose-1,6-bisphosphatase (FBPase), a rate-limiting enzyme in gluconeogenesis, has been shown to exert substantial influence on a range of tumorigenic processes. In humans, two isoforms of FBPase, namely FBP1 and FBP2, are present. FBP1 is ubiquitously expressed in diverse tissues, whereas FBP2 is exclusively localized in muscle tissue [6]. The metabolic function of FBP1 involves catalyzing the hydrolysis of fructose-1, 6-bisphosphate (F-1, 6-BP) into fructose-6-phosphate (F-6-P) and inorganic phosphate. FBP1 acts as a tumor suppressor in various cancer types. Specifically, FBP1 inhibits the potential Warburg effect by counteracting glycolytic flux in clear cell renal cell carcinoma [7], breast cancer [8–10], lung adenocarcinoma [11, 12], hepatocellular carcinoma [13, 14], and prostate cancer [15]. In basal-like breast cancer, the Snail-G9a-DNMT1 complex induces methylation of the FBP1 promoter, leading to down-regulation of FBP1. Consequently, this down-regulation reduces mitochondrial complex I activity, resulting in limited oxygen consumption and reduced production of reactive oxygen species (ROS). This metabolic reprogramming ultimately contributes to the development of enhanced cancer stem cell-like phenotypes [8]. Interestingly, recent studies have revealed the intriguing mechanism through which FBP1 exerts its tumor-suppressive effects in a catalytically inactive manner. Specifically, FBP1 interacts directly with the HIF inhibitory domain, thereby preventing the nuclear activity of hypoxia-inducible factors (HIFs) in clear cell renal cell carcinoma. This inhibition of HIFs subsequently impedes cell growth, glycolysis, and the pentose phosphate pathway [7]. Additionally, FBP1's binding to the WW domain of IQ motif containing GTPase-activating protein 1 (IQGAP1) blocks IQGAP1-dependent ERK1/2 phosphorylation, leading to an enhanced anticancer response to gemcitabine in pancreatic ductal adenocarcinoma [16]. FBP1 has been found to downregulate programmed death-1ligand (PD-L1) expression at the transcriptional level in pancreatic and prostate cancer, leading to an enhancement of tumor immunity. This regulatory mechanism is mediated by FBP1's inhibition of signal transducer and activator of transcription-3 (STAT3)-dependent PD-L1 transcription, as demonstrated in the previous studies [17]. Additionally, FBP1 has been shown to exhibit protein phosphatase activity, facilitating the dephosphorylation of IκBα. Consequently, this inhibits the activation of NF-κB and effectively suppresses colorectal tumorigenesis [18].

The transmembrane receptor Notch1, which has a molecular weight of 300 kDa, is activated by interacting with ligands expressed on neighboring cells. By sequential cleavage, the Notch intracellular domain (NICD) is released into the cytoplasm and translocate to the nucleus, thereby activating transcription of downstream target genes [19–21]. Thus, NICD is a key molecule to accomplish the downstream activation of Notch signaling. There are multiple mechanisms that govern the degradation of NICD, consequently impacting the activation of the Notch signaling pathway [22–28]. Recent research indicates that the activation of the Notch signaling pathway plays a role in the acquisition of properties associated with cancer stem cells (CSCs). Nuclear-Dbf2-related 1 (NDR1), through its competition with FBXW7 for binding to NICD1, diminishes the degradation of NICD1, resulting in the activation of the Notch pathway. This activation further facilitates the transcription of downstream target genes and contributes to the enrichment of breast CSCs [25]. The NUMB protein exerts a positive influence on the Notch signaling pathway, thereby preserving the stem cell characteristics of cortical neural progenitor cells. This effect is achieved through the facilitation of the interaction between the NICD1 and the deubiquitinating enzyme BAP1, resulting in the stabilization of NICD1 [27].

The current understanding of the roles of FBP1 in the maintenance of the cancer stem cell phenotype and the associated regulatory mechanisms is lacking. This study presents evidence that FBP1 plays a crucial role in regulating lung cancer stem cells, and that NICD1 is necessary for FBP1-mediated maintenance of the CSC phenotype. The underlying mechanism involves the interaction between FBP1 and NICD1, which facilitates the binding of the E3 ligase FBXW7 to NICD1. This, in turn, promotes the ubiquitination and destabilization of NICD1. Importantly, this regulatory mechanism is not dependent on the metabolic enzyme activity of FBP1. FBP1 is observed to be down-regulated in NSCLC, resulting in the hyperactivation of Notch signaling, thereby augmenting the CSC phenotype.

## Materials and methods

### Databases and data collection

RNA-sequencing expression (level 3) profiles and corresponding clinical information for NSCLC were obtained from The Cancer Genome Atlas (TCGA, https://portal.gdc.com). The mRNA stemness index (mRNAsi) was computed based on a previous study. The OCLR algorithm was employed to calculate the mRNAsi as proposed by Malta et al. [29]. The gene expression profile consisted of 11 774 genes based on the mRNA expression signature. Spearman correlation was utilized, and the minimum value was subtracted and divided by the maximum to map the stemness index within a specific range. Pearson correlations were computed to assess the relationship between FBP1 and stemness factors (ALDH1A1, KLF4, NANOG, OCT4A, and SOX2) in terms of mRNA expression levels. In this study, patients with NSCLC were categorized into two groups based on the expression level of FBP1: the Ntoch1 High group and the FBP1 Ntoch1 group. The overall survival (OS) of these two groups was compared using Kaplan–Meier curves. All the aforementioned analytical techniques and the R package were employed using the R foundation for statistical computing (2020) version 4.0.3.

### Cell lines and cell culture

The A549, H1299, and PC-9 human NSCLC cell lines were obtained from ATCC. A549 and H1299 cell lines were cultured in RMPI-1640 (Corning, New York, USA) supplemented with 10% FBS (Biological Industries, Kibbutz Beit-Haemek, Israel) in an incubator with 5% CO_2_ at 37 ℃. The PC-9 cell line was cultured in DMEM (Corning, New York, USA) supplemented with 10% FBS (Biological Industries, Kibbutz Beit-Haemek, Israel). Monthly PCR tests were conducted to confirm that all cell lines were free of contamination with Mycoplasma. Additionally, all cell lines used in the experiment had been passaged less than ten times.

### Plasmids transfection and lentivirus infection

The pCMV-MCS-3Flag vector was utilized to clone full-length FBP1 (Wild Type) and FBP1 (G260R mutant) cDNA, resulting in the generation of pCMV-FBP1(WT) and pCMV-FBP1(G260R) expression vectors, respectively. The plasmid sequences employed in the experiment were validated through sequencing. NSCLC cells were transfected with jetPRIME (Polyplus) according to the manufacturer's instructions. The specific shRNA sequence targeting FBP1 was as follows: 5′-AACATGTTCATAACCAGGTCG-3′. The control shNC was created using a nonsense oligonucleotide sequence. Lentiviral particles were generated by transfecting the shRNA plasmids into 293T cells. The viruses were harvested and applied to NSCLC cells. The FBP1 knockdown cells were selected using puromycin (2 μg/ml) for a duration exceeding 2 weeks.

### Quantitative real-time PCR (qPCR)

Total RNA was extracted using the Total RNA Extraction Kit (Solarbio, Beijing, China). Subsequently, 1000 ng of the total RNA was reverse transcribed into cDNA using the PrimeScript RT Master Mix Kit (Vazyme, Jiangsu, China). Quantitative real-time PCR was then conducted using the ChamQ Universal SYBR PCR Master Mix (Vazyme, Nanjing, China) and a StepOne Plus PCR system (Thermo Fisher, Massachusetts, USA). The quantitative real-time PCR employed the following primer pairs: Jagged1, 5′-GCCTCCTGTCGGGATTTGG-3′(forward) and 5′-AGTGATCGCCTGCATAGCCA-3′(reverse); Hes1, 5′-AAAAATTCCTCGTCCCCGGT-3′(forward) and 5′-GAATGCCGCGAGCTATCTTTCT-3′(reverse); Hey1, 5′-CGGACGAGAATGGAAACTTGA-3′(forward) and 5′-CTTGCTCCATTACCTGCTTCTC-3′(reverse); Nanog, 5′-CAATGGTGTGACGCAGGGATG-3′(forward) and 5′-CTGGCAGGAGAATTTGGCTGG-3′(reverse); OCT4A, 5′-ACCGAGTGAGAGGCAACCTG-3′(forward) and 5′-ACACTCGGACCACATCCTTCT-3′(reverse); ALDH1A1, 5′-ATGCTCACCCCACCTTCTTCA-3′(forward) and 5′-CAGTGGTAAGGTTTCTCACCTGT-3′(reverse); SOX2, 5′-GCGGAAAACCAAGACGCTCA-3′(forward) and 5′-CCGTTCATGTGCGCGTAACT-3′(reverse); GAPDH, 5′-ATGAATGGGCAGCCGTTAGG-3′(forward) and 5′-CCCAATACGACCAAATCAGAGAA-3′(reverse); GAPDH was used as an internal control.

### Western blot and Immunoprecipitation

Cells were lysed in RIPA buffer [50 mM Tris(pH 7.4), 150 mM NaCl, 1% NP-40, 0.5% sodium deoxycholate] supplemented with multiple protease inhibitor (Beyotime, Shanghai, China) and phosphatase inhibitor (Thermo Fisher Scientific, Massachusetts, USA). The protein was subsequently resolved using sodium dodecyl sulfate–polyacrylamide gel electrophoresis (SDS-PAGE) and transferred onto PVDF membranes (0.45 μm, Millipore, Massachusetts, USA). The membranes were then blocked using 1 × TBST containing 3% BSA and subsequently incubated overnight at 4 °C with the indicated primary antibodies. Following this, the membranes were washed five times with TBST and incubated with either goat anti-rabbit or goat anti-mouse antibody (Cell Signaling Technology, Massachusetts, USA) at room temperature for 1 h. The resulting conjugated antibody complexes were detected using ECL chemiluminescence substrate (Beyotime, Shanghai, China) and exposed to the Invitrogen iBright gel imaging system (Thermo Fisher Scientific, Massachusetts, USA) to obtain images. The gray values of each protein band relative to the internal reference were analyzed using the ImageJ software.

The immunoprecipitation (IP) assay was conducted following the instructions provided in the manual of the Crosslink Magnetic IP/Co-IP Kit (Thermo Fisher Scientific, Massachusetts, USA). In brief, the indicated primary antibody was conjugated to protein A/G magnetic beads through a 15-min incubation, and the antibody was cross-linked to the microbeads by incubating with DSS for 30 min. A negative control was established using normal mouse or rabbit IgG. The cell lysates were subjected to a 2-h incubation with the prepared microbeads at room temperature. Following two washes with wash buffer, the microbeads were eluted using elution buffer and subsequently boiled for 10 min to obtain immunoprecipitation (IP) samples.

### Measurement of fructose 1,6-bisphosphatase (FBPase) activity

FBPase activity was detected using a fructose 1,6-bisphosphatase activity assay kit (Solarbio, Beijing, China). Briefly, FBPase catalyzes the conversion of fructose-1,6-diphosphate and water into fructose 6-phosphate and inorganic phosphorus. Glucose phosphate isomerase and glucose 6-phosphate dehydrogenase were then added to the reaction system to catalyze the conversion of gluconic acid 6-phosphate and NADPH. FBPase activity was determined by measuring the rate of increase in NADPH at 340 nm using UV spectrophotometry.

### Flow cytometry analysis

Cells were seeded in 6-well plates at a density of 1 × 10^6^ cells per plate and allowed to incubate overnight. On the following day, the cells were digested using trypsin, centrifuged, and subsequently washed twice with phosphate-buffered saline (PBS). The culture medium, which contained the PE cross-linked CD133 fluorescent probe (Thermo Fisher Scientific, Massachusetts, USA), was then added and incubated for 30 min at a temperature of 37 °C. After incubation, the cells were washed twice with PBS and resuspended. The proportion of CD133-positive cells was determined using flow cytometry, with an excitation wavelength of 488 nm and an emission wavelength of 575 nm.

### CCK-8 assay

Cells were initially distributed into 96-well plates, followed by exposure to cisplatin at the specified concentrations after a 24-h interval. Subsequently, the cells were incubated at a temperature of 37 °C for a duration of 48 h, and their viability was assessed using a CCK-8 kit (Beyotime, Shanghai, China). The optical density (OD) value was measured at a wavelength of 450 nm.

### Sphere formation assay

To facilitate the generation of spherical structures, a concentration of 10,000 lung cancer cells per milliliter was cultivated in serum-free DMEM/F12 medium (Gibco, Massachusetts, USA) supplemented with 20 μg/ml epidermal growth factor (EGF) (Sigma-Aldrich, Massachusetts, USA), 10 μg/ml basic fibroblast growth factor (bFGF) (Sigma-Aldrich, Massachusetts, USA), and B27 supplement (Gibco, Massachusetts, USA) within ultra-low attachment 6-well plates (Corning, Massachusetts, USA). The culture medium was replenished with half its volume every 3 days, and the cells were allowed to grow for approximately 2 weeks until visible spheres were formed. The number of spheres was quantified using a microscope, and their diameters were measured by capturing images.

### Immunofluorescence

PC9 cells were initially cultured in Nunc Lab-Tek II chamber slides (Thermo Fisher Scientific, Massachusetts, USA). Following adherence, the cells were fixed using precooled absolute methanol for a duration of 10 min at room temperature. Subsequently, the fixed cells were permeabilized using a 1% Triton X-100 solution for 15 min. The cells were then subjected to a 30-min incubation with a blocking solution, followed by overnight incubation at 4 °C with FBP1 rabbit antibody and NICD1 mouse antibody (1:100 diluted). Afterward, the cells were incubated at room temperature for 1 h with Alexa Fluor 488 preadsorbed goat anti-rabbit antibody and APC preadsorbed goat anti-mouse antibody. Finally, the fluorescent images were captured using a laser scanning confocal microscope (Flowview FV3000, Olympus, Japan). Colocalization of the two fluorescences was analyzed using Image-Pro Plus software (Version 6.0.0).

### Animal studies

To establish the xenograft tumor model, indicated numbers (10^4^, 10^3^, and 10^2^) of H1299 cells were subcutaneously injected into NOD/SCID mice (male, 6 weeks old, 22–24 g). The tumor volumes were measured every 3 days and calculated as 1/2 × length × width^2^. At the end of experiment, the tumors were carefully removed, counted, weighed, and photographed. The frequency of CSCs was calculated by ELDA software [30]. All NOD/SCID mice were maintained and used in accordance with the guidelines of Laboratory Animal Centre of Zhejiang University School of Medicine First Affiliated Hospital.

## Results

### Analysis of the TCGA database revealed a significant correlation between reduced expression of FBP1 and elevated mRNA stemness index in non-small cell lung cancer (NSCLC)

**Fig. 1:**
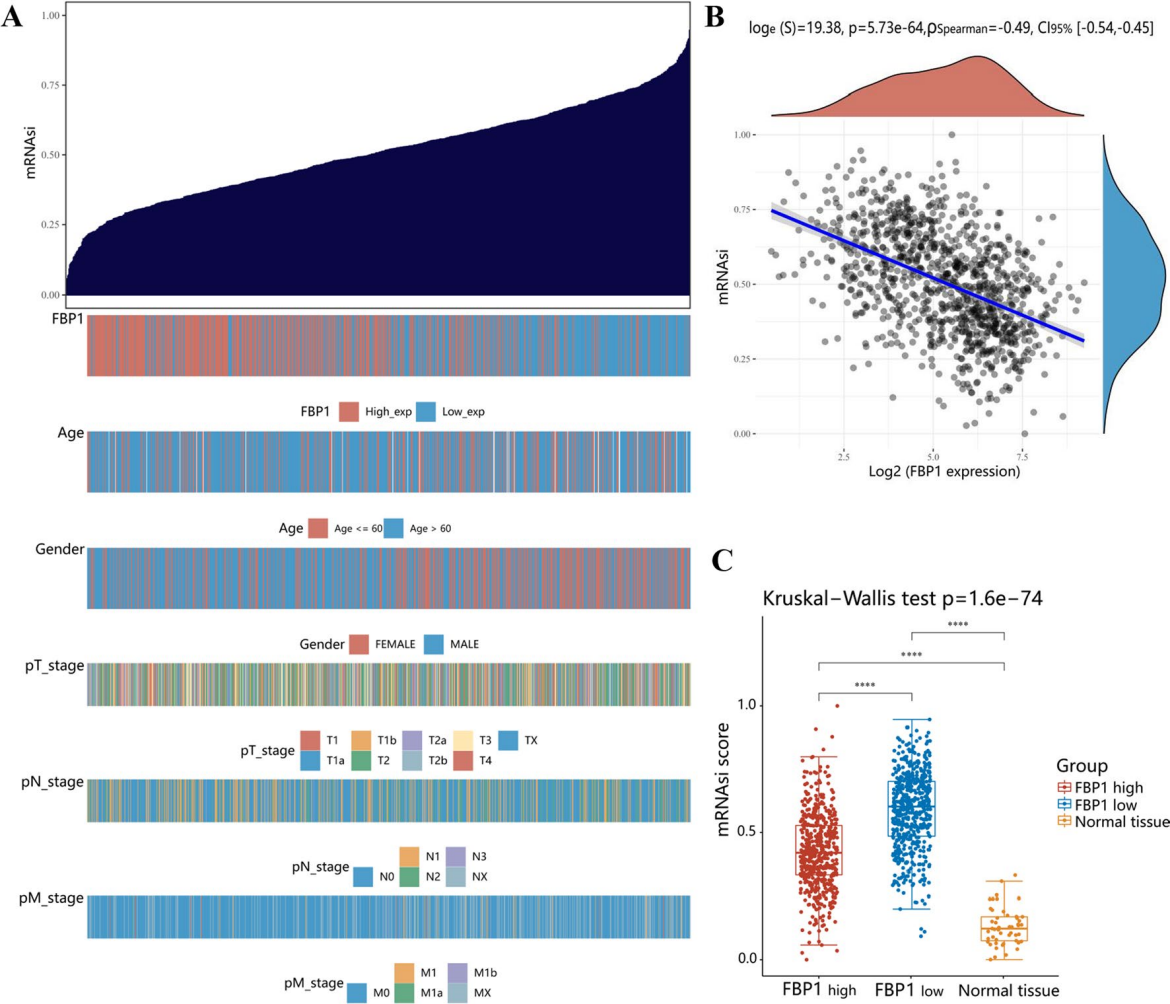
The mRNA stemness index model demonstrated a negative correlation between FBP1 and stemness in non-small cell lung cancer (NSCLC).** A** The distribution map of mRNA stemness index (mRNAsi) score and clinical information. The top figure depicting the distribution of mRNAsi score from low to high, and the bottom figure illustrating the distribution of FBP1 expression level and clinical information characteristics after sorting. **B** Spearman correlation analysis of mRNAsi score and FBP1 expression. The density curve on the right illustrates the distribution trend of the mRNAsi score, while the upper density curve represents the distribution trend of gene expression. The value displayed on the top indicates the correlation p value, correlation coefficient, and the method used for correlation calculation. **C** The distribution of mRNAsi scores in FBP1 high expression NSCLC group, FBP1 low expression NSCLC group, and Normal tissue group. ****p < 0.0001. The Wilcox test was used to compare the statistical difference between two groups, while the Kruskal–Wallis test was employed to test the significance difference among the three groups

Our previous investigation confirmed the down-regulation of FBP1 in lung adenocarcinoma, where it functioned as a tumor suppressor by inhibiting proliferation and epithelial–mesenchymal transition (EMT) in lung adenocarcinoma cells [31]. Accumulating research findings support a direct association between EMT and the preservation of cancer stem cell (CSC) characteristics [32–36]. To enhance our comprehension of FBP1 in NSCLC, we conducted an investigation into the role of FBP1 in maintaining the phenotype of cancer stem cells. We acquired the RNA-sequencing expression profile and relevant clinical data of 1 017 NSCLC patients (including lung squamous cell carcinoma and lung adenocarcinoma) from the TCGA database. Subsequently, we utilized the OCLR algorithm, developed by Malta et al. [29], to compute the mRNA stemness index (mRNAsi) for each sample (Fig. 1A). Upon comparing the mRNAsi with the expression level of FBP1 and the clinical characteristics of the patients, we discovered that a higher mRNAsi was linked to a lower FBP1 expression, as well as advanced age (> 60 years), female gender, and advanced pTNM stage (Fig. 1).

### FBP1 was found to suppress the cancer stem cell phenotype of NSCLC

**Fig. 2:**
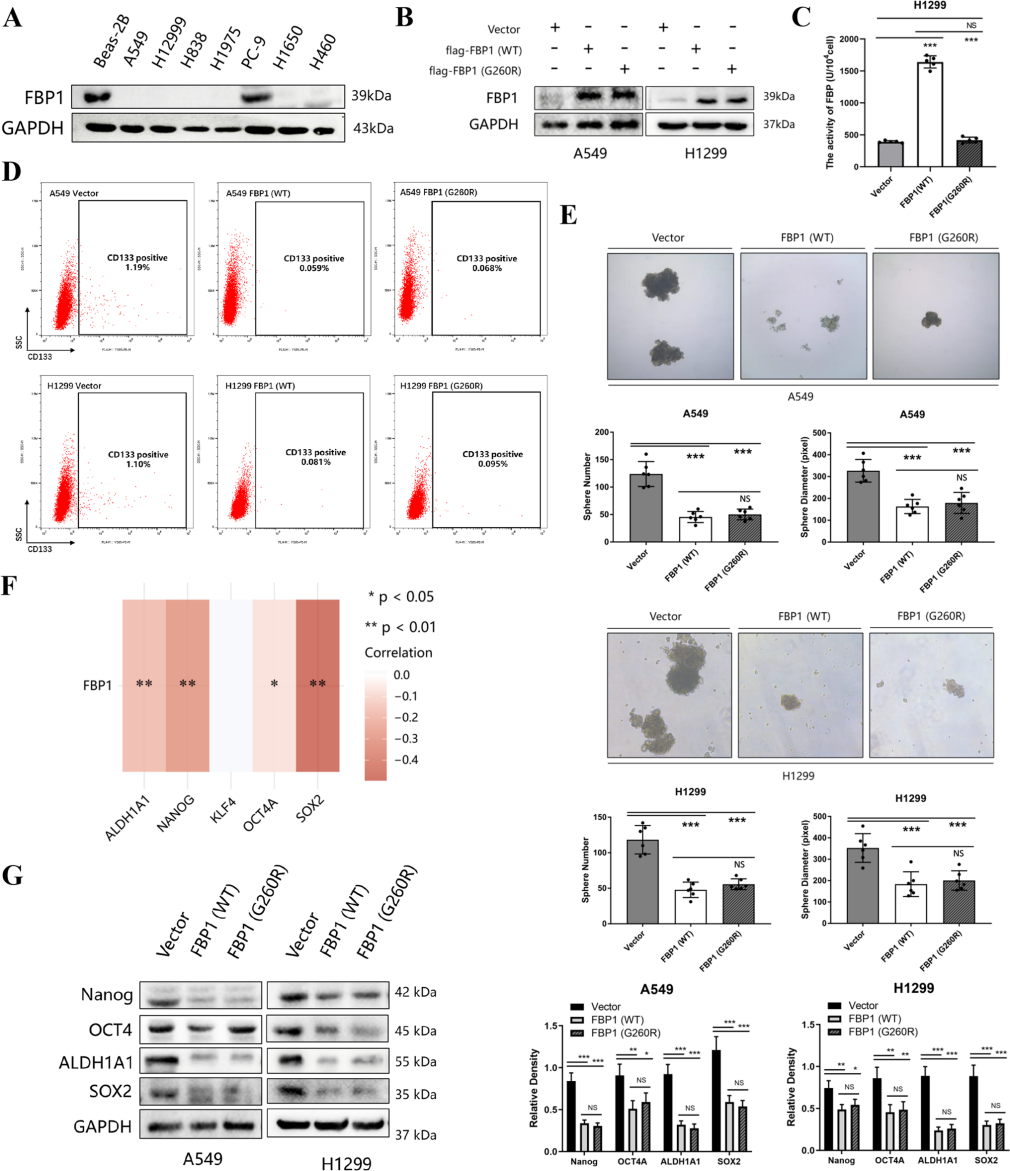
Overexpression of exogenous FBP1 impairs the cancer stem cell phenotype in NSCLC. A549 and H1299 cells were infected with lentivirus carrying vector, FBP1 (wild type, WT), or FBP1 (G260R mutation, G260R) for stable overexpression. **A** The expression levels of FBP1 in different tumors were examined using TCGA database. **B** The efficiency of FBP1 overexpression in A549 and H1299 cells was assessed by Western blot analysis. **C** The FBPase activity of the blank vector, FBP1 wild-type (WT) and FBP1 G260R mutant variants was detected using a FBPase activity assay kit in H1299 cells. **D** Viable A549 and H1299 cells were stained with CD133 dye, and the proportion of CD133-positive cells was determined using flow cytometry. **E** Representative images of A549 and H1299 tumor spheres were observed under a microscope at 40X magnification. **P < 0.01. ***P < 0.001. **F** The relationship between FBP1 and stemness factors at the mRNA level was investigated using the TCGA dataset. **G** The expression levels of stemness factors in A549 and H1299 cells were determined through Western blot analysis. *NS* No significant difference. *P < 0.05. **P < 0.01. ***P < 0.001

Further validation was performed in NSCLC cell lines. Initially, the protein levels of FBP1 were compared between NSCLC cell lines (A549, H1299, H838, H1975, PC9, H1650, and H460) and a normal bronchial epithelial cell line (Beas-2B). It was observed that FBP1 was down-regulated in most NSCLC cells, except for PC9, when compared to the normal bronchial epithelial cell (Fig. 2A). To investigate the necessity of FBP1's metabolic enzymatic activity for its ability to inhibit stemness, flag-tagged FBP1 plasmids were constructed with a G260R point mutation, rendering the metabolic enzymatic activity inactive, based on previous research [7]. A549 and H1299 cells were chosen for introducing exogenous FBP1 overexpression, including both wild-type (WT) and G260R mutant variants. The efficacy of FBP1 expression was assessed using Western blot analysis (Fig. 2B). The enzymatic activity of FBP1 G260R mutant variants in H1299 cells was detected to be in the inactive state (Fig. 2C). CD133, a well-established surface marker for lung cancer stem cells [37–39], was utilized to determine the proportion of CD133-positive cells in A549 cells through flow cytometry. Intriguingly, the results indicated that the overexpression of either wild-type or G260R mutant FBP1 led to a decrease in the proportion of CD133-positive cells compared to the control group. Furthermore, there was no significant difference observed between the overexpression of FBP1 (G260R) and FBP1 (WT) (Fig. 2D). Following this, we conducted an examination to ascertain the impact of FBP1 overexpression on the in vitro ability of tumor spheres formation. The overexpression of both FBP1 (WT) and FBP1 (G260R) resulted in a hindered sphere formation ability in A549 and H1299 cells, as evidenced by a reduction in the quantity and size of tumor spheres (*P* < 0.001, Fig. 2E). The resistance to chemotherapy drug is a critical characteristic of cancer stem cells (CSCs) and is the primary cause of their recurrence following chemotherapeutics [40]. To investigate this, we subjected A549 and H1299 cells to varying concentrations of cisplatin and assessed cell viability using the CCK8 assay. The findings of the study indicated that the overexpression of FBP1 (WT) resulted in a significant decrease in the IC50 value of cisplatin treatment. Conversely, there was no significant difference observed between FBP1 (G260R) and the FBP1 (WT) group (Supplemental Fig. 1A). A549 and H1299 cells overexpressed with FBP1 demonstrated a notably elevated proportion of apoptosis and necrotic cells subsequent to a 48-h cisplatin treatment, in comparison to the control cells (Supplemental Fig. 1B). Additionally, an analysis was conducted to investigate the mRNA level correlation between FBP1 and stemness markers (ALDH1A1, Nanog, KLF4, OCT4a, and SOX2) in NSCLC, utilizing the TCGA database. The results revealed a significant negative correlation between FBP1 and most stemness markers (*P* < 0.05), with the exception of KLF4 (Fig. 2F). Consequently, the impact of FBP1 overexpression on stemness markers at the protein level was further examined. Consistent with the previous findings, both FBP1 (WT) and FBP1 (G260R) were found to inhibit the expression of ALDH1A1, Nanog, OCT4a, and SOX2 (Fig. 2G). The aforementioned findings suggest that the overexpression of FBP1 negatively affects the cancer stem cell characteristics of NSCLC, regardless of the metabolic enzyme activity of FBP1.

**Fig. 3:**
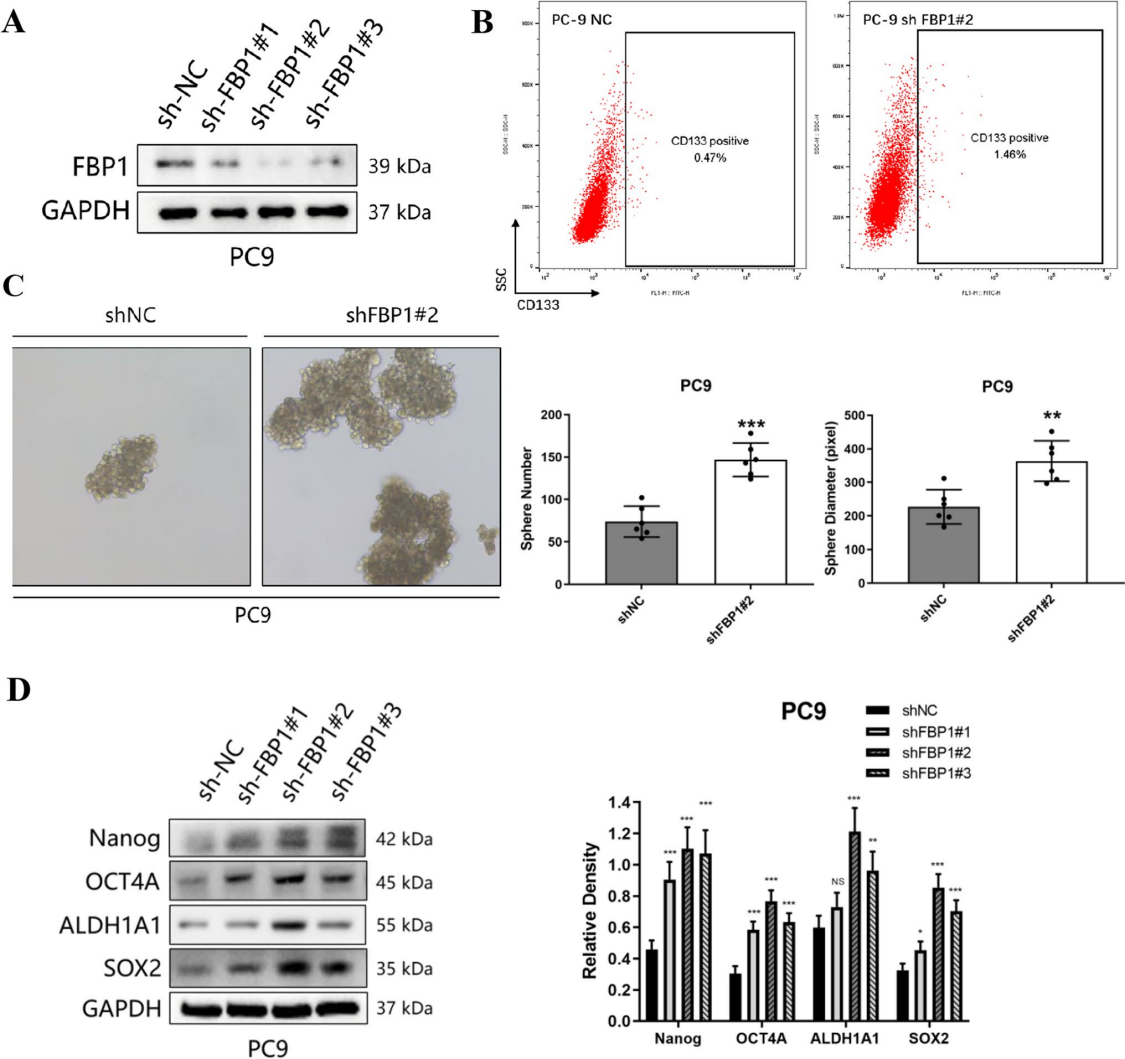
Knockdown of FBP1 enhanced the cancer stem cell phenotype of NSCLC. PC9 cell was infected with lentivirus carrying vector or shFBP1 for stable overexpression. **A** The efficiency of FBP1 knockdown in the PC9 cell was assessed using Western blot analysis. **B** The proportion of CD133-positive cells was determined through flow cytometry. **C** Microscopic images of PC9 tumor spheres were captured at a magnification of 40X. **P < 0.01. ***P < 0.001. **D** The expression levels of stemness factors were evaluated in PC9 cell. *NS* No significant difference. *P < 0.05. **P < 0.01. ***P < 0.001

To further investigate the role of FBP1 in mediating the cancer stem cell phenotype, we generated stable knockdown PC9 cells with reduced FBP1 expression (Fig. 3A). The knockdown of FBP1 resulted in an increased proportion of CD133-positive cells in PC9 (Fig. 3B). Additionally, the knockdown of FBP1 enhanced the capacity for tumor sphere formation, as evidenced by an increased number of spheres (*P* < 0.001) and larger sphere diameter (*P* < 0.01, Fig. 3C). Furthermore, the inhibition of FBP1 led to an increased resistance of PC9 cells to cisplatin (*P* < 0.05, Supplemental Fig. 1C). After a 48-h treatment with cisplatin, the FBP1 knockdown group exhibited a significantly lower proportion of necrotic cells in PC9 cells compared to the control group (Supplemental Fig. 1D). Consistent with previous observations, the knockdown of FBP1 also led to an increase in the protein expression levels of stemness markers (Fig. 3D).

### FBP1 inhibits the activation of NOTCH1 and its downstream pathways

**Fig. 4:**
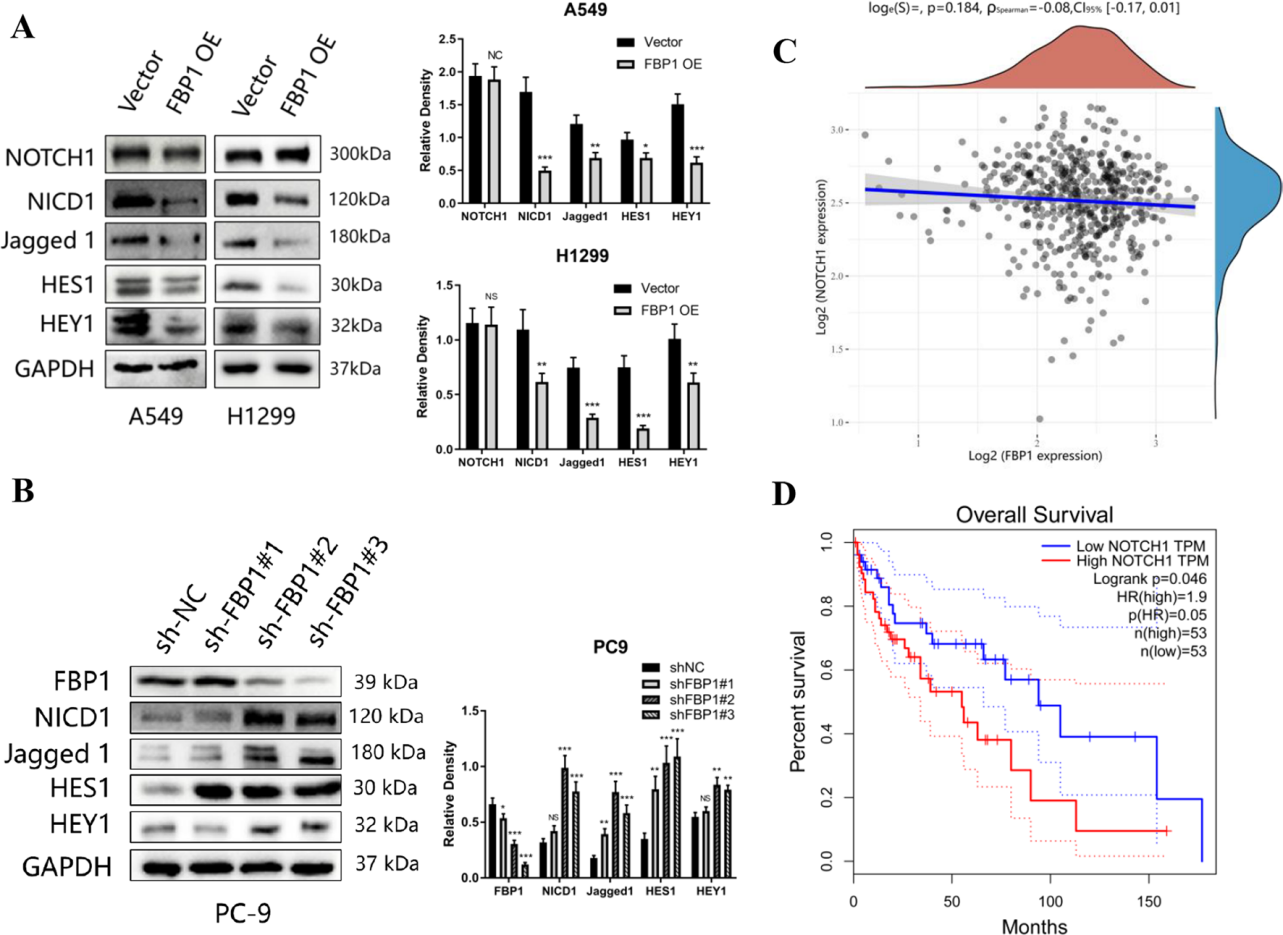
FBP1 inhibits the activation of NOTCH1 and its downstream pathways. **A**, **B** A549 and H1299 cells were infected with either vector or FBP1 overexpression lentivirus, while PC9 cells were infected with either vector or shFBP1 lentivirus. The expression levels of NOTCH1 pathway markers were determined using Western blot analysis. **C** Additionally, the correlation between FBP1 and NOTCH1 expression levels in NSCLC was analyzed using the TCGA database. **D** Kaplan–Meier analysis was performed to assess overall survival based on the expression level of NOTCH1

To investigate the mechanism through which FBP1 regulates the CSC phenotype in NSCLC, we examined the impact of FBP1 overexpression or knockdown on the Notch1 signaling pathway, which is widely acknowledged to be involved in the regulation of stemness. Western blot analysis of the expression levels of Notch1 intracellular domain (NICD1), its ligand Jagged1, and its downstream target Hes1/Hey1 revealed that the overexpression of FBP1 in A549 and H1299 cells resulted in the down-regulation of markers associated with the Notch1 signaling pathway (Fig. 4A). The results obtained from qPCR analysis demonstrated that the overexpression of FBP1 resulted in a decrease in the mRNA expression levels of jagged1, Hes1, and Hey1. However, there was no significant impact observed on the mRNA expression level of Notch1 (Supplemental Fig. 1E). Moreover, the expression levels of FBP1 and NICD1, along with the downstream markers of the NOTCH pathway, were detected at various time intervals subsequent to the infection of A549 and H1299 cells with FBP1 overexpression lentivirus. The findings revealed a significant increase in FBP1 expression at 48-h post-lentivirus infection compared to 24 h post-infection. Conversely, the protein level of NICD1 at 48-h post-lentivirus infection was notably lower than that at 24 h post-infection, and the expression levels of downstream markers of the NOTCH pathway were also diminished (Supplemental Fig. 1F). In PC9 cells, the depletion of FBP1 resulted in elevated levels of associated proteins (Fig. 4B). Conversely, the overexpression of FBP1 did not induce notable alterations in the expression of pathway markers within the Hedgehog pathway, which is also implicated in the regulation of cancer stem cell phenotype (Supplemental Fig. 1G). Intriguingly, an examination of mRNA expression data from the TCGA database revealed no significant transcriptional correlation between FBP1 and NOTCH1 in NSCLC (Fig. 4C). Furthermore, our findings substantiate a significant correlation between elevated NOTCH1 expression and unfavorable prognosis in NSCLC, as indicated by the Kaplan–Meier survival analysis results (Fig. 4D). Additionally, we conducted a protein-level assessment of NICD1 and Jagged1 in both a normal alveolar epithelial cell line (Beas-2B) and seven NSCLC cell lines (Supplemental Fig. 1H).

### The impairment of the NOTCH signaling pathway is a crucial factor in the suppression of the lung cancer stem cell phenotype by FBP1

**Fig. 5:**
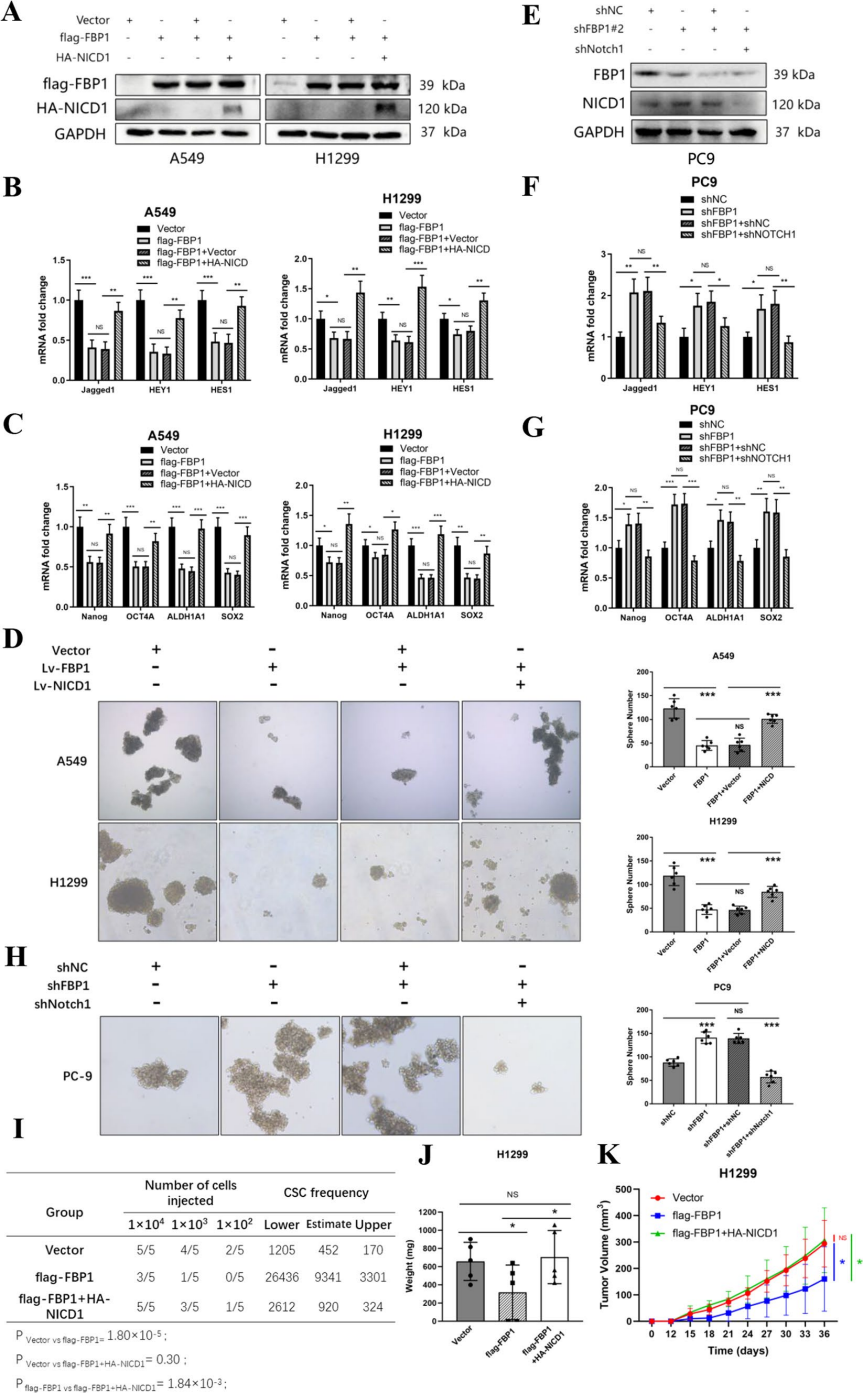
NICD1 plays a pivotal role in the inhibition of NSCLC stemness by FBP1. A549 and H1299 cells were transfected with either a vector, flag-FBP1 plasmid, or HA-NICD1 plasmid for a duration of 48 h. Similarly, PC9 cells were transfected with a vector, shFBP1#2 plasmid, or shNotch1 plasmid for the same duration. **A**, **E** The transfection efficiency of flag-FBP1 and HA-NICD1 plasmids (**A**) and knock down efficiency of FBP1 (**E**) were confirmed through Western blot analysis. **B**, **F** qPCR was employed to assess the expression levels of downstream markers of the NOTCH1 pathway in A549, H1299 cells (**B**), and PC9 cells (**F**). **C**, **G** qPCR was utilized to determine the expression levels of stemness transcriptional factors (**C**, **G**). **D**, **H** Representative images of A549, H1299 (**D**), and PC9 (**H**) tumor spheres were captured under a microscope at 40X magnification. NS: No significant difference. ***P < 0.001. **I** Tumor incidence and CSCs frequency were calculated using limiting dilution analysis. **J** The weight of subcutaneous tumors in the three groups of mice injected with 10^4^ H1299 cells was measured on day 36. *NS* No significant difference. *P < 0.05. **K** Tumor volume growth curves of three groups of mice injected with 10^4^ H1299 cells. *NS* No significant difference. *P < 0.05. Blue line: vector vs flag-FBP1; Green line: flag-FBP1 vs flag-FBP1 + HA-NICD1; Red line: vector vs flag-FBP1 + HA-NICD1

To validate the critical role of NICD1 in FBP1-mediated suppression of the CSC phenotype, A549 and H1299 cells were subjected to overexpression of flag-tagged FBP1 (WT) or flag-tagged FBP1 (WT) with HA-tagged NICD1 (Fig. 5A). The overexpression of flag-tagged FBP1 (WT) resulted in a significant transcriptional inhibition of Notch1 signaling pathway markers (*P* < 0.05). However, the introduction of HA-tagged NICD1 effectively reversed this trend (Fig. 5B). In a similar manner, the up-regulation of flag-tagged FBP1 (WT) resulted in a down-regulation of stemness transcription factors (Nanog, OCT4a, ALDH1A1 and SOX2) at the mRNA level (*P* < 0.05). However, this effect was reversed when HA-tagged NICD1 was co-overexpressed (Fig. 5C). This trend was also observed at the protein level (Supplemental Fig. 2A, B). Furthermore, in the sphere formation assay, the number of tumor spheres formed in A549 and H1299 cells with stable overexpression of FBP1 (WT) was significantly lower compared to the control group (*P* < 0.001). Nevertheless, the number of tumor spheres was restored in A549 and H1299 cells co-overexpressing NICD1 (Fig. 5D).

To assess the impact of FBP1 and NICD1 on the CSCs frequency, an in vivo limiting dilution analysis was conducted. The results revealed a noteworthy decline (*P* = 1.80 × 10^–5^) in tumor formation when H1299 cells overexpressed FBP1 (WT) in comparison to the control group. However, the co-overexpression of HA-NICD1 significantly augmented (*P* = 1.84 × 10^–3^) tumorigenicity when compared to the sole overexpression of flag-FBP1 (WT) (Fig. 5I and Supplemental Fig. 2C). In contrast to mice that were subcutaneously injected with 10^4^ cells individually, the overexpression of FBP1 led to a reduction in tumor weight and growth rate, whereas the overexpression of NICD1 promoted tumor weight and growth rate (*P* < 0.05, Fig. 5J*, *K).

In contrast, we conducted knockdown experiments on FBP1 or both FBP1 and Notch1 in PC9 cells (Fig. 5E). The knockdown of FBP1 resulted in a significant enhancement of Notch signaling pathway transcriptional activation (Fig. 5F) and an increase in the expression of stemness transcription factors at the mRNA level (Fig. 5G). Conversely, the simultaneous knockdown of Notch1 counteracted the activation of the Notch signaling pathway (Fig. 5F) and reduced the expression of stemness factors (Fig. 5G) at the mRNA level. This trend was also observed at the protein level (Supplemental Fig. 2D, E). The knockdown of FBP1 led to a significant increase in the formation of tumor spheres in PC9 cells, while the simultaneous knockdown of Notch1 reversed this trend (Fig. 5H).

### FBP1 facilitates the degradation of NICD1 through the ubiquitin–proteasome pathway, independent of its enzymatic activity

**Fig. 6:**
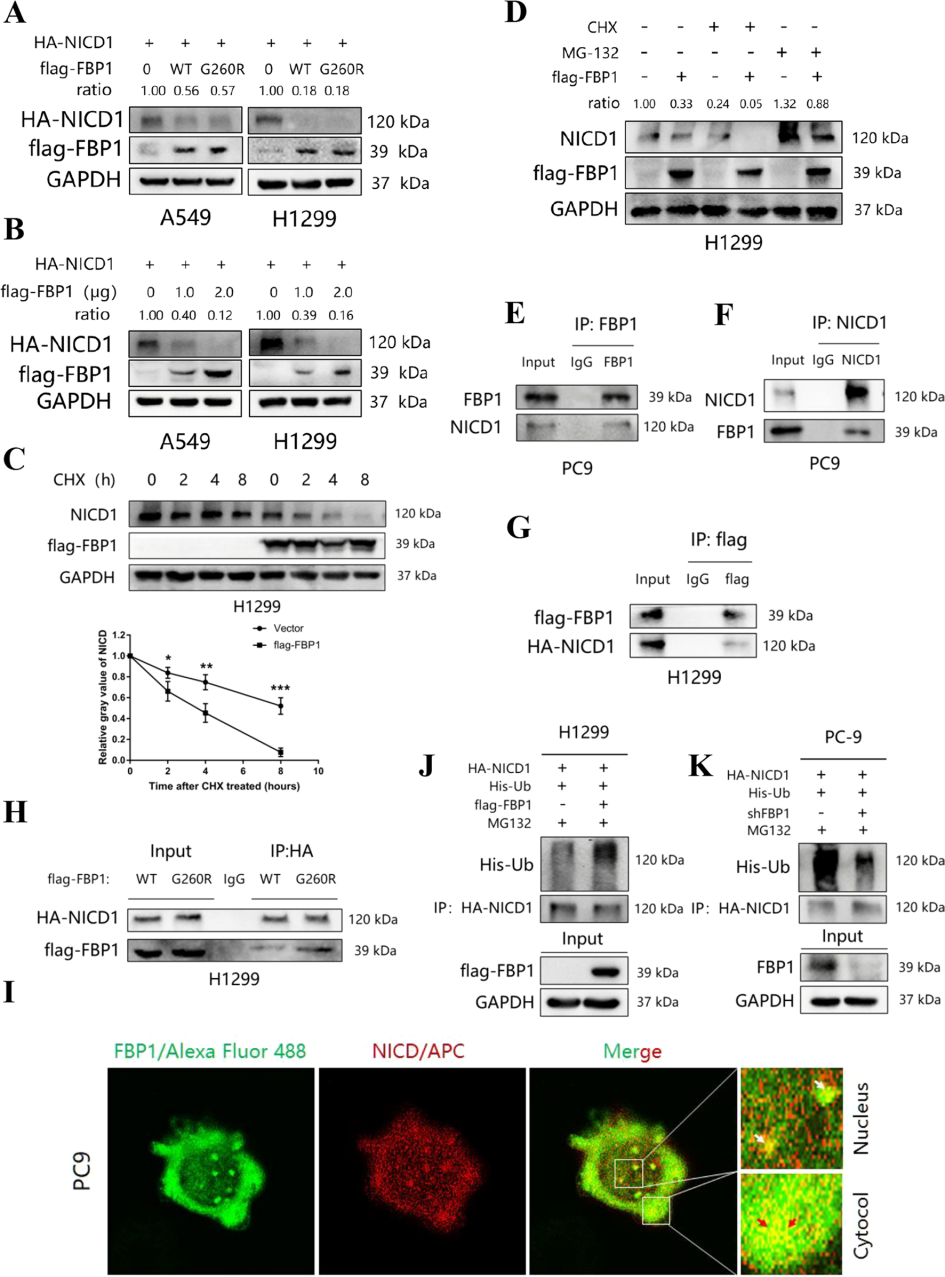
FBP1 promotes NICD1 degradation via the ubiquitin–proteasome pathway**. A** A549 and H1299 cells were transfected with 1 μg HA-NICD1 plasmid and co-transfected with either 1 μg flag-FBP1 (wild type, WT) plasmid or flag-FBP1(G260R mutation, G260R) plasmid for a duration of 48 h. The expression level of HA-NICD1 was then assessed using Western blot analysis. **B** A549 and H1299 cells were transfected with 1 μg HA-NICD1 plasmid and co-transfected with either 0.5 μg or 1 μg flag-FBP1 (WT) plasmid for 48 h, and the expression level of HA-NICD1 was evaluated using Western blot analysis. **C** H1299 cells were transfected with 1 μg of either Vector or flag-FBP1(WT) plasmid for a duration of 48 h, followed by treatment with 100 mg/ml cyclohexane (CHX) for varying time intervals. The degradation half-life of endogenous NICD1 was assessed using Western blot analysis. *P < 0.05. **P < 0.01. ***P < 0.001. **D** H1299 cells were transfected with 1 μg of either Vector or flag-FBP1(WT) plasmid for a duration of 48 h, followed by treatment with 100 mg/ml cyclohexane (CHX) for 8 h or 20 mg/ml MG-132 for 8 h. The expression level of endogenous NICD1 was determined using Western blot analysis. **E–F** Co-immunoprecipitation (Co-IP) assay was conducted to demonstrate the interaction between endogenous FBP1 and NICD in PC9 cells. **(G)** H1299 cells were transfected with 1 μg of flag-FBP1 and 1 μg of HA-NICD1 plasmids for a duration of 48 h. The Co-IP assay revealed the interaction between exogenous FBP1 and NICD in H1299 cells. **H** H1299 cells were transfected with 1 μg of HA-NICD1 plasmids and either 1 μg of flag-FBP1(WT) plasmid or flag-FBP1(G260R) plasmid for a duration of 48 h. Subsequently, the Co-IP assay demonstrated the interaction between exogenous FBP1 and NICD1 in H1299 cells. **I** A549 cells were subjected to staining with fluorescent antibodies targeting FBP1 and NICD1, respectively. The colocalization of FBP1 and NICD1 in cells was observed using a confocal microscope. **J**, **K** H1299 **(J)** and PC9 **(K)** cells were subjected to transfection with the specified plasmids for a duration of 48 h, followed by treatment with MG-132 for a period of 8 h. The quantification of NICD1's ubiquitination level was conducted through Co-IP assay

As FBP1 does not exert control over Notch1 expression at the transcriptional level, we investigated whether FBP1 influences post-translational modifications of NICD1. A549 and H1299 cells were transfected with 1 μg of HA-NICD1 plasmid and co-transfected with either 1 μg of flag-FBP1(WT) plasmid or flag-FBP1(G260R) plasmid. Surprisingly, both the wild-type FBP1 and the G260R mutant FBP1 resulted in a reduction of NICD1 expression at the protein level (Fig. 6A). Additionally, A549 and H1299 cells were subjected to transfection with 1 μg HA-NICD1 plasmid and concomitantly transfected with either 0.5 μg or 1 μg flag-FBP1(WT) plasmid. It was observed that the overexpression of flag-FBP1(WT) at higher concentrations led to a further reduction in the protein level of HA-tagged NICD1 (Fig. 6B). Moreover, an investigation was conducted to determine the impact of FBP1 on the degradation of endogenous NICD1. H1299 cells were transfected with either 1 μg Vector or flag-FBP1(WT) plasmid and subsequently treated with 100 mg/ml cyclohexane (CHX) for varying durations. The findings demonstrated that the overexpression of FBP1 significantly shortened the half-life of endogenous NICD1 in H1299 cells (*P* < 0.05, Fig. 6C). To investigate whether the enhancement of NICD1 degradation induced by FBP1 is mediated through the proteasome pathway, H1299 cells were transfected with 1 μg Vector or flag-FBP1(WT) plasmid and subsequently treated with 100 mg/ml CHX for 8 h or 20 mg/ml proteasome inhibitor MG-132 for 8 h. Notably, the degradation of endogenous NICD1 was significantly accelerated following CHX treatment, whereas the protein level of endogenous NICD1 was significantly higher compared to the control group after MG-132 treatment (Fig. 6D). To confirm the protein–protein interaction between FBP1 and NICD1, a coimmunoprecipitation (CO-IP) assay was performed.

The study observed the interaction between endogenous and exogenous FBP1 and NICD1 (Fig. 6E–H). To ascertain where FBP1 and NICD1 interact in the cell, cytosolic and nuclear proteins were isolated and the expression levels of FBP1 and NICD1 were assessed in the cytoplasm and nucleus, respectively. The findings revealed that FBP1 predominantly localized in the cytoplasm, whereas NICD1 exhibited comparable expression levels in both the cytoplasm and nucleus (Supplemental Fig. 2F). Additionally, the localization of FBP1 and NICD1 in PC9 cells was determined using an immunofluorescence assay, confirming their colocalization in both cytoplasm and nucleus (Fig. 6I). H1299 cells were transfected with HA-NICD1, His-Ub, flag-FBP1, or Vector plasmids and treated with MG132. Immunoprecipitation (IP) analysis revealed a significant increase in NICD1 ubiquitination levels when FBP1 was overexpressed compared to the control group (Fig. 6J). Conversely, knockdown of FBP1 resulted in a significant reduction in NICD1 ubiquitination levels compared to the control group (Fig. 6K).

### FBXW7 is the prominent E3 ubiquitin ligase recruited by FBP1 to facilitate the degradation of NICD1

**Fig. 7:**
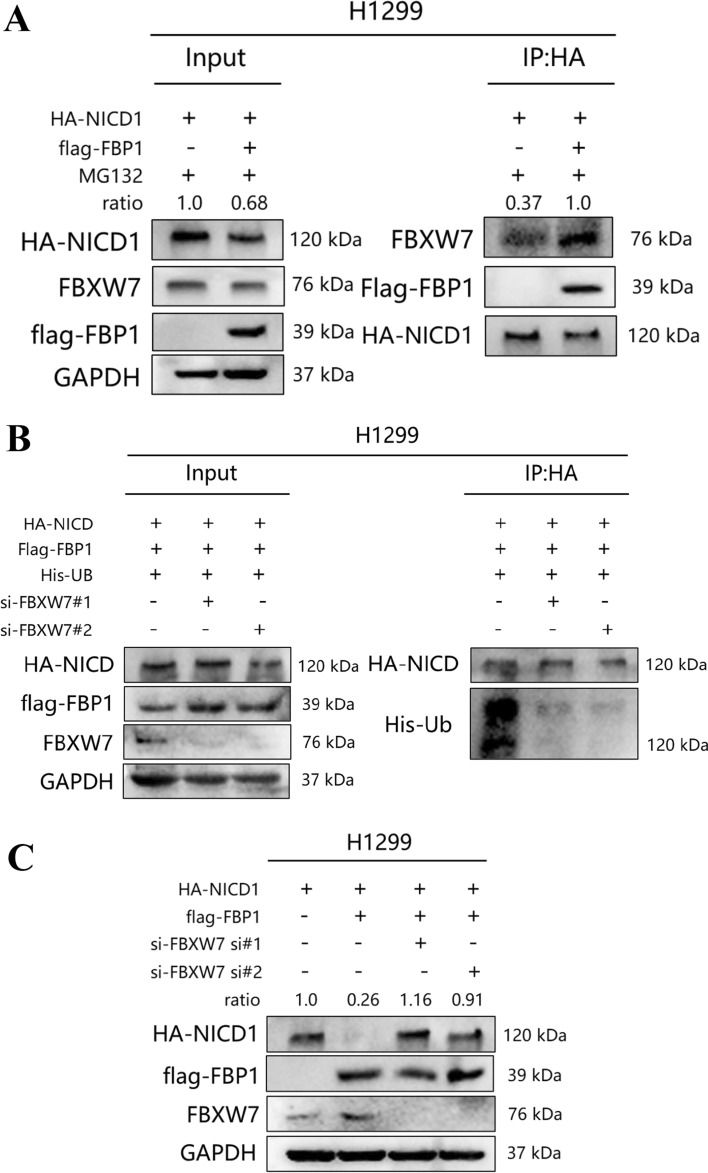
Identification of the E3 ligase recruited by FBP1 in relation to NICD1. **A** H1299 cells were transfected with the designated plasmids for 48 h and subsequently treated with MG-132 for 8 h to determine the E3 ligase that interacts with FBP1 and NICD1 via CO-IP assay. **B** In H1299 cells, transfection with specific plasmids was conducted for a duration of 48 h. Western blot analysis was employed to ascertain alterations in the ubiquitination levels of NICD1 subsequent to the knockdown of FBXW7 using siRNAs. **C** Changes in the expression levels of NICD1 were determined through Western blot analysis following the knockdown of FBXW7 using siRNAs in H1299 cells transfected with the indicated plasmids for 48 h

FBXW7 plays a crucial role as a tumor suppressor and is among the most frequently observed ubiquitin–proteasome system proteins in human cancer [41]. Multiple studies have confirmed its involvement in the degradation of NICD, prompting our investigation into whether FBXW7 is necessary for FBP1-mediated NICD1 degradation [23, 25, 26]. To explore this, H1299 cells were transfected with HA-tagged NICD1, flag-tagged FBP1 (WT) plasmids, or Vector plasmids, and subsequently treated with MG-132. Through CO-IP assay, it was determined that overexpression of flag-FBP1 led to increased binding between NICD1 and FBXW7 (Fig. 7A).

Subsequently, FBXW7 was silenced in H1299 cells, and a CO-IP assay confirmed that FBXW7 knockdown led to a decrease in NICD1 ubiquitination when flag was overexpressed (Fig. 7B). Ultimately, it was discovered that the overexpression of flag-tagged FBP1 notably down-regulated the protein level of NICD1, while the simultaneous knockdown of FBXW7 reversed this trend (Fig. 7C). Furthermore, it was observed that the deubiquitinating enzyme USP12 played a role in the regulation of NICD1 ubiquitination. The overexpression of USP12 in H1299 cells significantly mitigated the heightened NICD1 ubiquitination level caused by FBP1 overexpression (Supplemental Fig. 3).

## Discussion

FBP1 plays a crucial role in the regulation of gluconeogenesis and constrains glycolytic flow, thus frequently exhibiting underexpression in various cancer types as a tumor suppressor [7, 11, 13, 42]. Prior investigations have demonstrated that FBP1 is down-regulated in NSCLC as a consequence of abnormal methylation occurring within its promoter DNA sequence [11, 42, 43]. The restoration of FBP1 expression in lung adenocarcinoma effectively hindered cell proliferation and invasion under hypoxic conditions in vitro, while also suppressing tumor growth in vivo [42]. The inhibition of FBP1 by promoter methylation mediated by GBE1 leads to the up-regulation of HIF1α levels through the NF-κB signaling pathway, resulting in the activation of anaerobic glycolysis and increased glucose uptake [11]. Despite these findings, the specific mechanisms by which FBP1 regulates CSC properties have not been extensively investigated.

The Notch signaling pathway is widely recognized as a highly conserved mechanism that plays a crucial role in numerous biological processes, including organogenesis and tissue regeneration [44]. Following a three-step enzymatic cleavage, the Notch intracellular domain (NICD) is ultimately released. Upon translocation into the nucleus, the RAM region of NICD binds to CBF-1/suppressor of hairless/Lag1 (CSL) proteins. This interaction between NICD and CSL protein leads to the conversion of the "cooperative inhibition complex" into a "cooperative activation complex", subsequently forming a polyprotein–DNA complex with DNA to initiate the activation of relevant genes [44]. Dysregulated NOTCH signaling has been implicated as an oncogenic driver in various cancers [45]. Despite not being widely recognized as a "stemness factor" in the conventional sense, mounting evidence suggests that the activation of the Notch signaling pathway promotes the preservation and enlargement of stem cell populations, particularly in glioma [46, 47], breast cancer [25, 48, 49], ovarian cancer [50, 51], lung cancer [52, 53], and other solid tumors [27]. The up-regulation of Notch in ovarian cancer results in the expansion of CSCs and heightened resistance to platinum-based therapies. Conversely, the inhibition of the Notch pathway through the use of γ-secretase inhibitors (GSI) can diminish CSCs and enhance tumor responsiveness to platinum drugs [50]. Similarly, our findings substantiate the crucial involvement of NICD1 in FBP1-mediated regulation of stemness (Fig. 5).

Post-translational modifications exert regulatory control over the stability and activity of NICD. Specifically, phosphorylation of the C-terminal PEST domain of NICD by cyclin C/Cdk8 triggers the recognition and ubiquitination of NICD by an E3 ligase complex comprising FBXW7, thereby promoting the proteasomal degradation of NICD [54, 55]. In the context of breast cancer, FBP1 augments the ubiquitination of Notch1 via the Fbw7 pathway, subsequently leading to its degradation by the proteasome [28]. The promotion of the binding between NICD1 and the deubiquitinating enzyme BAP1 by NUMB, resulting in the inhibition of NICD1 ubiquitination and the subsequent stabilization of NICD, is a well-established phenomenon. BAP1 is known to interact with various domains of NUMB, including the PRR region, NICD1 RAM-ANK, and PEST domains [27].

**Fig. 8:**
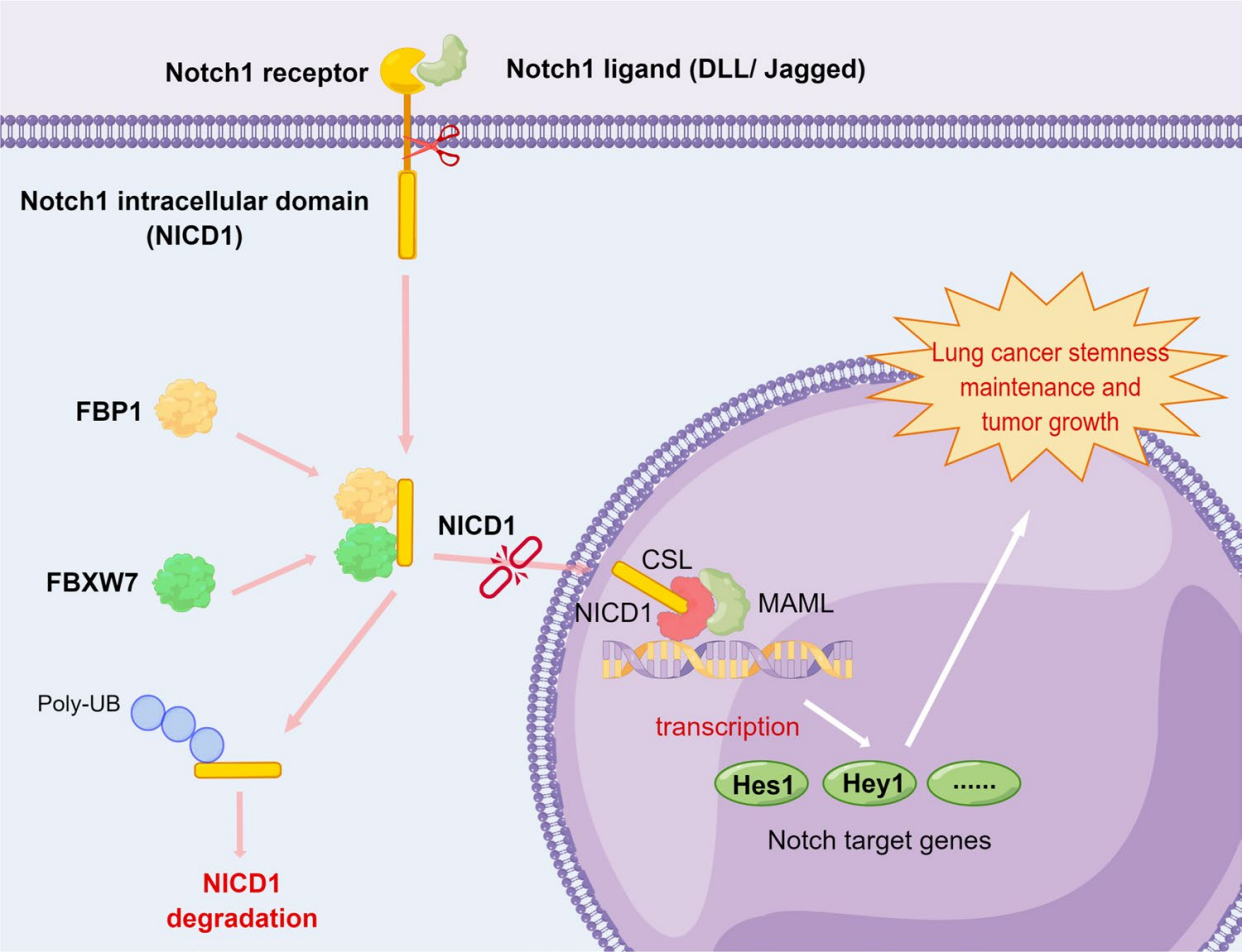
A schematic model underlying the role of the FBP1–FBXW7–NICD1 axis in cancer stem cell phenotype of NSCLC. *DLL* delta-like, *UB* Ubiquitin, *CSL* CBF-1/suppressor of hairless/Lag1, *MAML* mastermind-like protein, *HES1* hairy/enhancer of Split-1, *HEY1* Hairy/enhancer of split related to YRPW motif-1

In our study, we have discovered that FBP1 plays a crucial role as a partner in the interaction between the NICD1 and E3 ligase FBXW7. This interaction enhances the binding of FBXW7 to NICD1, leading to a reduction in NICD1 protein stability through the ubiquitin–proteasome pathway (Fig. 8). These findings are consistent with the previous research in this field [28]. The FBP1–FBXW7–NICD1 pathway is responsible for the inhibition of Notch signaling activation and the suppression of stem cell-like properties in NSCLC by FBP1. Notably, the metabolic enzyme activity of FBP1 does not necessary for this regulatory mechanism. However, further investigation is required to determine the specific role of FBP1's non-enzymatic activity and the corresponding domains involved.

In summary, these findings unveil a novel mechanism by which FBP1 functions in NSCLC and suggest that it could be a potential target for eliminating the recurrence and drug resistance characteristics of CSCs.

### Supplementary Information

Below is the link to the electronic supplementary material.

Supplementary file1 (PDF 920 KB)

## Data Availability

Data for this study can be obtained by contacting the corresponding author by email after obtaining consent from the corresponding author.
