# Supplementary figures and images for: FBP1 inhibits NSCLC stemness by promoting ubiquitination of Notch1 intracellular domain and accelerating degradation
Source: Cell Mol Life Sci. 2024 Feb 13;81(1):87. doi: 10.1007/s00018-024-05138-x (PMC10864425; doi:10.1007/s00018-024-05138-x)

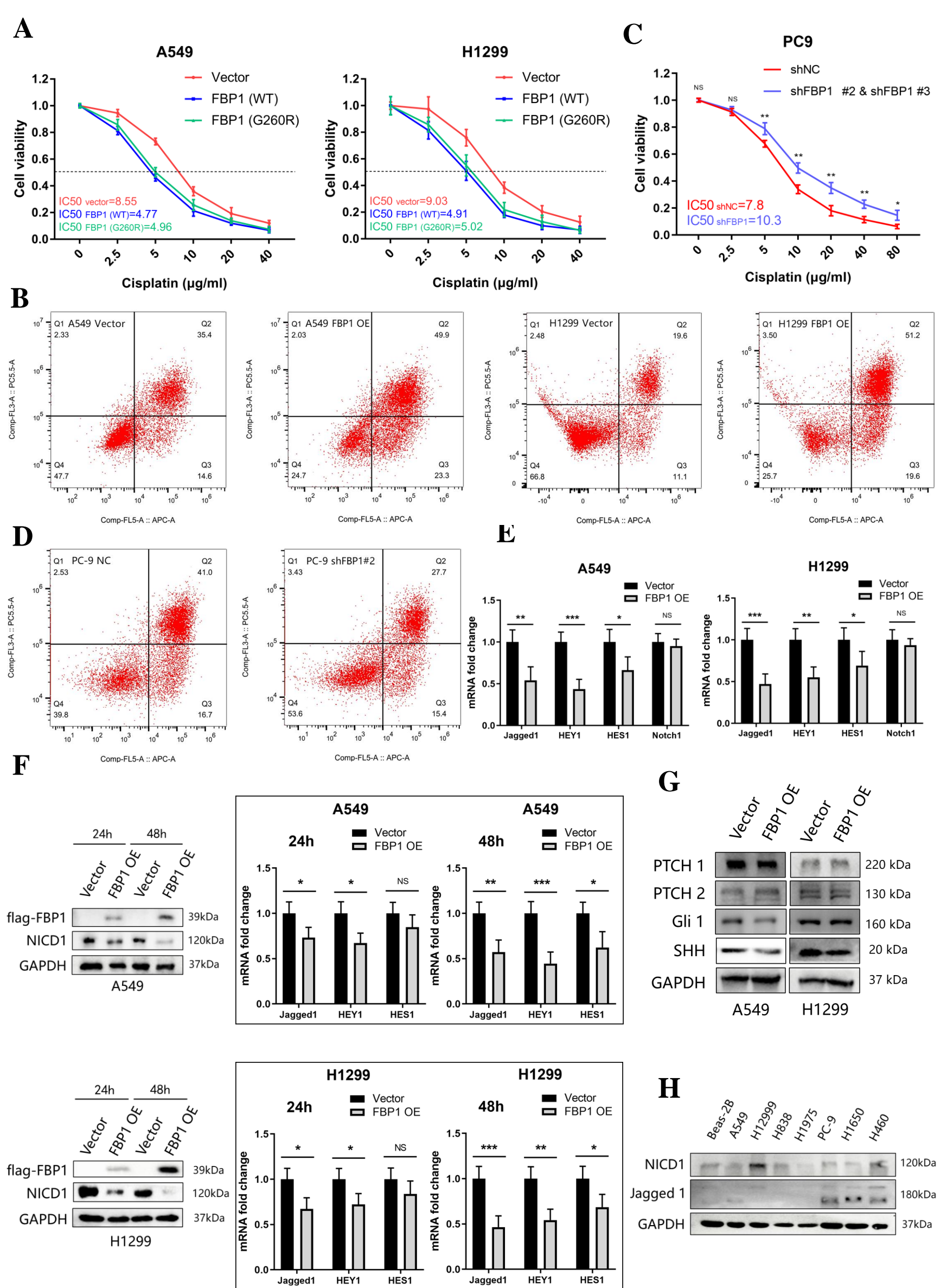

**A**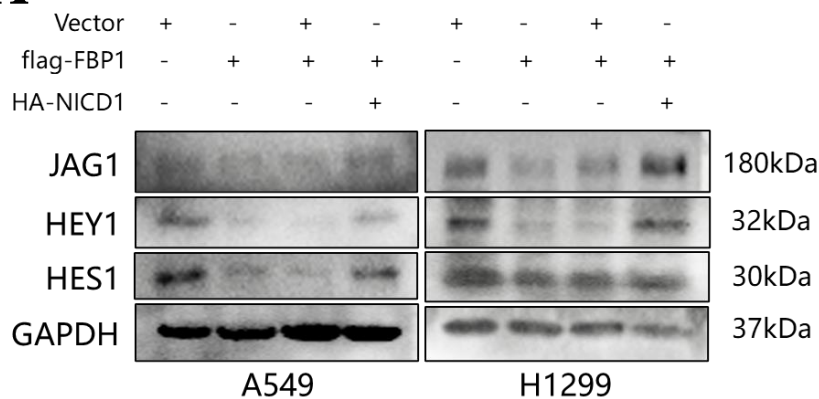**B**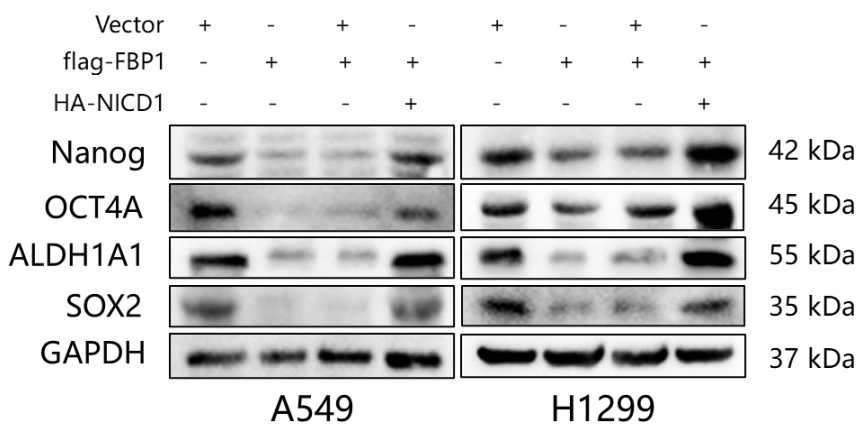**C**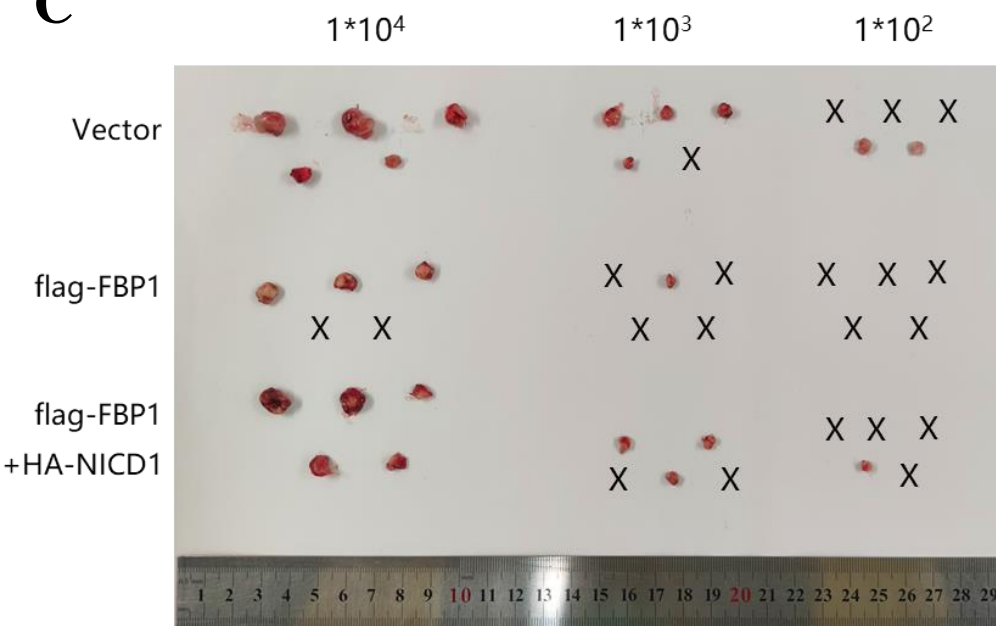**D**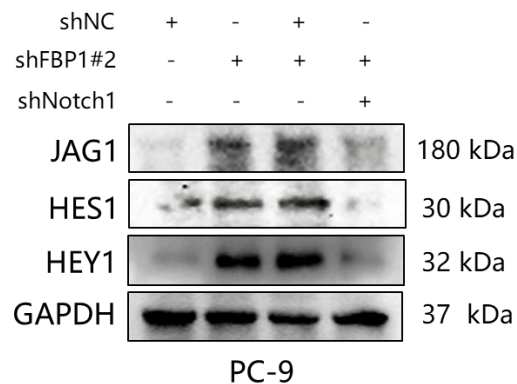**E**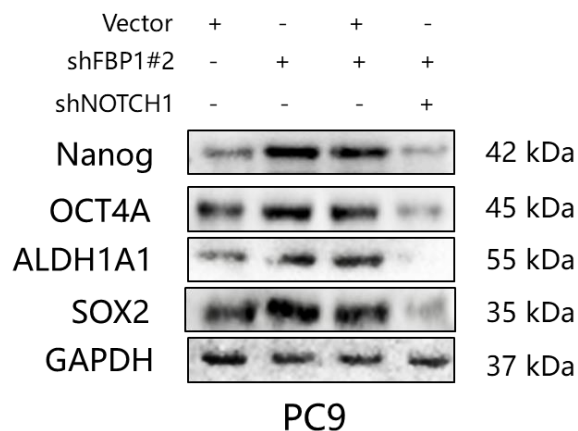**F**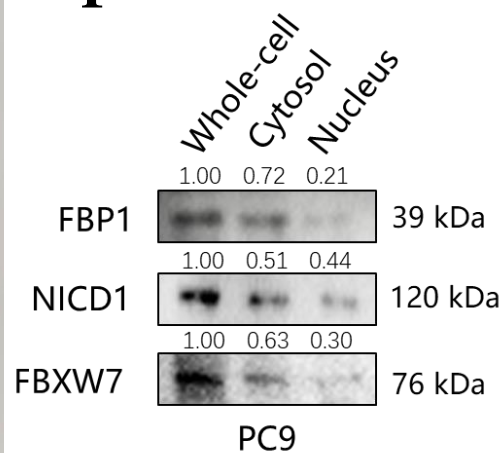

# H1299

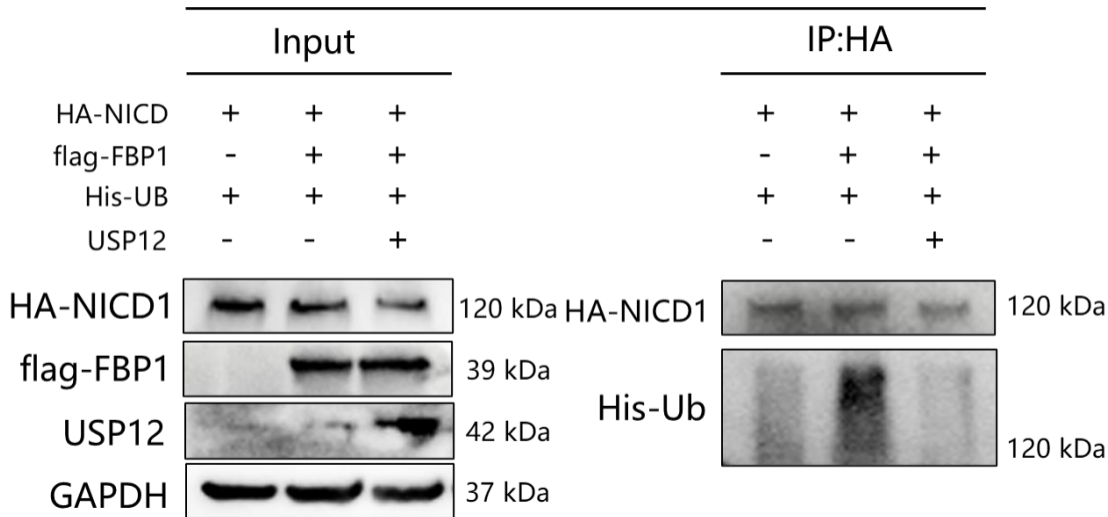

Supplement: Supplementary file 1 — Supplementary file1 (PDF 920 KB) [file 18_2024_5138_MOESM1_ESM.pdf]
